# Phylogeography and Demographic History of Chinese Black-Spotted Frog Populations (*Pelophylax nigromaculata*): Evidence for Independent Refugia Expansion and Secondary Contact

**DOI:** 10.1186/1471-2148-8-21

**Published:** 2008-01-24

**Authors:** Hua Zhang, Jie Yan, Guoqiang Zhang, Kaiya Zhou

**Affiliations:** 1 Jiangsu Key Laboratory for Biodiversity and Biotechnology, College of Life Sciences, Nanjing Normal University, Nanjing 210046, China

## Abstract

**Background:**

Pleistocene glaciations had considerable impact on phylogeographic patterns within and among closely related species of many vertebrates. Compared to Europe and North America, research on the phylogeography of vertebrates in East Asia, particularly in China, remains limited. The black-spotted frog (*Pelophylax nigromaculata*) is a widespread species in East Asia. The wide distribution of this species in China makes it an ideal model for the study of palaeoclimatic effects on vertebrates in East Asia. Our previous studies of *P. nigromaculata *revealed significant subdivisions between the northeast China populations and populations in other regions of the mainland. In the present study, we aim to see whether the deepest splits among lineages and perhaps subsequent genealogical divisions are temporally consistent with a Pleistocene origin and whether clade geographic distributions, with insight into expansion patterns, are similarly spatially consistent with this model.

**Results:**

Using 1143 nucleotides of the mitochondrial cytochrome *b *gene from 262 individuals sampled from 28 localities, two main clades (clade A and clade B) differing by *c*. 7.72% sequence divergence were defined from parsimony analyses. The corresponding timing of lineage divergence, 0.92 Mya, indicates a most likely Pleistocene split. The A clade is further subdivided into two sub-clades, A1 and A2 with 1.22% sequence divergence. Nested clade phylogeographical and population demographic analyses suggested that the current distribution of this frog species was the result of range expansion from two independent refugia during the last interglacial period. We discovered a population within which haplotype lineages A and B of *P. nigromaculata *coexist in the Dongliao area of China by nucleotide sequences, PCR-RFLP and ISSR (inter simple sequence repeat) patterns. The ISSR result in particular supported divergence between the mitochondrial clades A and B and implied introgressive gene flow between the two divergent lineages.

**Conclusion:**

Nested clade phylogeographical and population demographic analyses indicate that the current distribution of *P. nigromaculata *is the result of range expansion from two independent refugia during the last interglacial period in late Pleistocene. One refugium was in east China and the lower elevations of south-western plateau. The distribution of the other mitochondrial clade is consistent with the presence of a refugium in the Korean Peninsula. The gene flow as detected by ISSR markers suggests a range expansion of the two refugia and a secondary contact between the two highly divergent lineages in the Dongliao (DL) area of northeast China.

## Background

During Pleistocene glaciations, Northwest Europe, Siberia and the northern most regions in North America were covered with ice sheets [1,2]. Pleistocene glaciations had extensive impact on phylogeographic patterns within and among closely related species of many vertebrates [3]. In Europe and North America, various species have been studied to determine the climatic and geological effects on phylogeographic patterns and population structures. In Europe and America, species dispersed to southern locations to survive in refugia and then expanded northward again during interglacial periods when climate recovered to or exceeded current mean global temperatures. Although there are discussion and debates regarding the degree of Pleistocene effects on speciation in North American birds [4-6], Pleistocene conditions appear to have played an active role both in initiating intraspecific and species-level divergences of the most closely related North American taxa [7]. However, climate recovery in East Asia during interglacial periods did not seem to resemble that of Europe or North America primarily due to the continuing uplift of the Tibet Plateau during late Tertiary. In China, the glacial advance was not as extensive as in Europe and North America because of the monsoons in East Asia. Biotic zones of Asia were also located at higher northern latitude than on other continents [8-10]. Despite the relatively mild climate, species' distribution were affected by climatic fluctuations during Pleistocene. However, research on the phylogeography of vertebrates in East Asia, especially in China, remains limited both in terms of the number of model species and geological timescales.

The black-spotted frog (*Pelophylax nigromaculata*) is a widespread species found in East Asia covering both Palaearctic and Oriental realms. The wide distribution of this species in Northeast, North, East, Central and Southwest China renders it an ideal model for investigating the palaeoclimatic effects on vertebrates in East Asia. *P. nigromaculata *is variable in size and color pattern over its wide geographic range; but shows no distinct morphological differentiation among populations and no subspecies have been designated. Traditionally, the black-spotted frog was treated as a member of genus *Rana*, but was recently placed into *Pelophylax*, a genus reintroduced by Fei *et al*. [11]. Recent studies on the phylogeny of ranid frogs based on mitochondrial and nuclear DNA sequences consistently supported *Pelophylax *as a well resolved monophyletic unit [12-15].

Studies of molecular phylogeography of *P. nigromaculata *have been independently reported by two groups. Yang *et al*. proposed that during the last interglacial period, *P. nigromaculata *experienced a rapid population expansion and that its distribution range fluctuated latitudinally in response to climatic oscillations [16]. But details of the evolutionary history and population structure were not inferred because of the small number of sampling localities (eight) and the relatively few Cyt *b *sequence data (704 bp). In a previous study, we examined the genetic structure of this frog in mainland China using 5'control region sequences and detected significant subdivision between Jilin-Liaoning (Northeast China) populations and other mainland populations [17].

**Figure 1:**
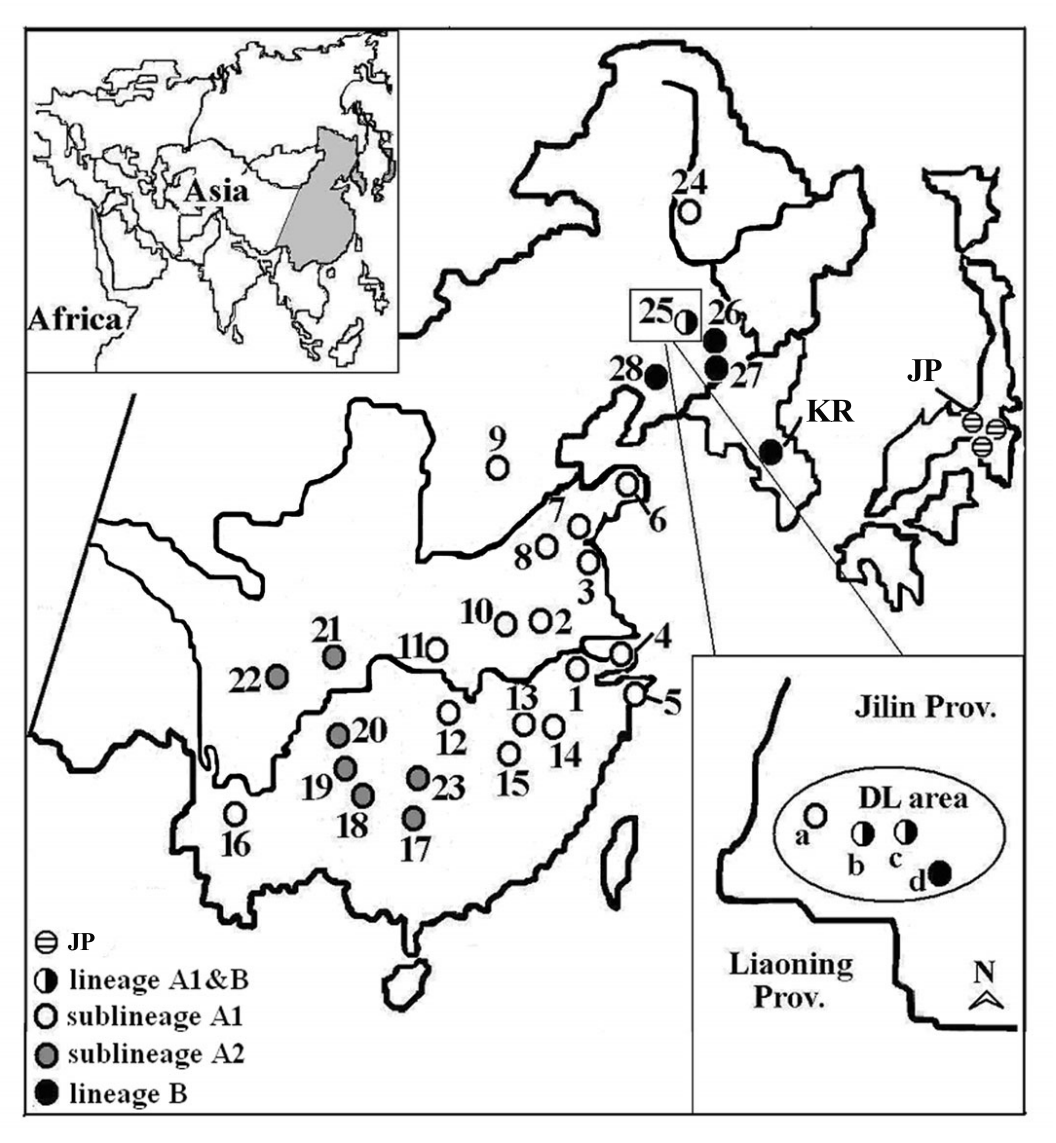
**Sampling sites and mitochondrial haplotype distributions of *P. nigromaculata *populations**. The numbers indicate local populations (see Table 1 for details). KR and JP stand for haplotypes from Korea and Japan, respectively. Lower case letters "a" to "d" indicate distinct but geographically proximate locales within the Dongliao area.

In this report, we tested the hypothesis that the Quaternary glaciations were the causes of this significant genetic structure. We analyzed the complete mitochondrial Cyt *b *sequences and generated ISSR fingerprint data in order to reconstruct the phylogeographic patterns in black-spotted frog populations sampled from 28 localities across the Chinese range (Fig. 1, Table 1). These data allowed us to assess the evolutionary history (demography, age and origins) of various Chinese populations and to address the hypothesis that the observed phylogeographic divisions are consistent with past range fragmentation by Pleistocene glaciations.

**Table 1:** Collection data for *P. nigromaculata *specimens included in this study.

Locality	County, Province (Abbr.)	n	Clade	Haplotype no.	Coordinates	GenBank Accession No.
1	Guangde, Anhui (GD)	10	A	32–40	30°53'N; 119°24'E	AY803813–AY803817, AY803831, DQ006243–DQ006245
2	Yuexi, Anhui (YX)	4	A	98–100	30°52'N; 116°22'E	AY803818–AY803820
3	Ganyu, Jiangsu (GU)	5	A	77, 106–109	34°50'N; 119°07'E	AY803821–AY803825
4	Cixi, Zhejiang (CX)	10	A	5–12	30°11'N; 121°15'E	AY803826–AY803829, DQ006233–DQ006236
5	Xiangshan, Zhejiang (XS)	6	A	118	29°29'N; 121°51'E	AY803830
6	Laiyang, Shandong (LA)	4	A	110–113	36°58'N; 120°42'E	AY803832–AY803835
7	Linyi, Shandong (LI)	4	A	114–116	35°03'N; 118°20'E	AY803836–AY803838
8	Zaozhuang, Shandong (ZZ)	10	A	52, 89–97	34°52'N; 117°33'E	AY803839–AY803843, DQ006263–DQ006267
9	Luquan, Hebei (LQ)	8	A	52–57	38°02'N; 114°29'E	AY803843–AY803847, DQ006251
10	Huangchuan, Henan (HC)	10	A	3, 41–48, 77	32°07'N; 115°02'E	AY803823, AY803848–AY803852, DQ006246–DQ006249
11	Shashi, Hubei (SS)	10	A	43, 70–77	30°18'N; 112°16'E	AY803823, AY803851, AY803853–AY803855, DQ006258–DQ006261
12	Nanxian, Hunan (NX)	10	A	53, 62–68, 77	29°22'N; 112°23'E	AY803844, AY803856–AY803858, DQ006253–DQ006256
13	Gao'an, Jiangxi (GA)	9	A	24–31	28°25'N; 115°22'E	AY803859–AY803861, DQ006238–DQ006241
14	Yiyang, Jiangxi (YY)	4	A	86–88	28°24'N; 117°26'E	AY803862–AY803863, AY803881
15	Xiajiang, Jiangxi (XJ)	5	A	83–86	27°33'N; 115°08'E	AY803864–AY803866, AY803881
16	Baoshan, Yunnan (BS)	6	A	1–4	25°08'N; 99°10'E	AY803850, AY803867–AY803869
17	Guilin, Guangxi (GL)	8	A	101–103	25°17'N; 110°17'E	AY803870–AY803872
18	Dushan, Guizhou (DS)	8	A	20–23	25°49'N; 107°32'E	AY803873–AY803874, AY803877, DQ006237
19	Guiyang, Guizhou (GY)	4	A	22, 103–105	26°35'N; 106°42'E	AY803872, AY803874–AY803876
20	Renhuai, Guizhou (RH)	5	A	20	27°49'N; 106°24'E	AY803877
21	Dazhu, Sichuan (DZ)	5	A	20, 117	30°45'N; 107°11'E	AY803877–AY803878
22	Guanghan, Sichuan (GH)	10	A	20	30°58'N; 104°15'E	AY803877
23	Wugang, Hunan (WG)	6	A	20, 81–82	26°43'N; 110°37'E	AY803877, AY803879–AY803880
24	Qiqiha'er, Heilongjiang (QQ)	7	A	14, 19, 69	47°20'N; 123°57'E	AY803883–AY803884, DQ006257
25	Dongliao, Jilin (DL)	12	A	13–19	42°55'N; 124°59'E	AY803882–AY803887, AY803889
25-a	Dongliao, Jilin (DL)	12	A	--	42°58'N; 124°52'E	--
25-b	Dongliao, Jilin (DL)	10	A, B	--	42°55'N; 124°59'E	--
25-c	Dongliao, Jilin (DL)	15	A, B	--	42°55'N; 125°01'E	--
25-d	Dongliao, Jilin (DL)	19	B	--	42°52'N; 125°11'E	--
26	Liuhe, Jilin (LH)	10	B	15, 49–51	42°16'N; 125°44'E	AY803888–AY803890, DQ006250
27	Tonghua, Jilin (TH)	8	B	15, 78–80	41°43'N; 125°56'E	AY803889, AY803891–AY803893
28	Liaoyang, Liaoning (LY)	18	B	15, 58–61	41°16'N; 123°12'E	AY803889, AY803894–AY803895, DQ006252

For each population sampled (alphanumeric locality codes refer to those in Fig. 1), we list collection localities (province, county, and exact coordinates), sample sizes (n), the unique mtDNA haplotype numbers present, GenBank accession numbers, and the clade (A, B) represented.

## Results

### Cytochrome *b *variation

Of the entire 1143 bp of Cyt *b *sequences, 197 nucleotides are variable and 130 are parsimony-informative. Nine, 29, and 159 variable sites are in the first, second, and third codon positions, respectively. No indels or premature stop codons were observed. This result, together with a strong bias against guanine (mean G = 13.3%, A = 25.8%, T = 27.4% and C = 33.4%), implies that the target fragment is mitochondrial Cyt *b *rather than its nuclear homologue. We identified 118 haplotypes among the 216 *P. nigromaculata *complete Cyt *b *sequences [GenBank: AY803813~AY803895 and DQ006233~DQ006267]. Eighty-seven of these were unique haplotypes, 19 were shared within local populations and 12 were shared among local populations (Table 2). The sequence distances (based on the HKY model) vary from 0.09 to 8.65% (mean 2.2%) among ingroup taxa, distances among different haplotype clades vary from 1.10% to 7.73% (Table 3). The overall haplotype diversity (h) and nucleotide diversity (π) were 0.975 and 0.028, respectively.

**Table 2:** Distribution of Cyt *b *haplotypes shared in different local populations of *P. nigromaculata*.

	BS	HC	DL	QQ	LH	LY	TH	DS	DZ	RH	GH	WG	GY	SS	LQ	ZZ	NX	XJ	YY	GU	GL
**3 (A1)**	2	1																			
**14 (A1)**			3	2																	
**19 (A1)**			1	4																	
**43 (A1)**		1												1							
**52 (A1)**															2	1					
**53 (A1)**															1		1				
**77 (A1)**		1												2			1			1	
**86 (A1)**																		1	1		
**20 (A2)**								5	4	5	10	3									
**22 (A2)**								1					1								
**103 (A2)**													1								1
**15 (B)**			2		6	2	3														

Rows: haplotypes and the lineages they belong to; columns: populations. The integers correspond to the number of individuals found with each haplotype.

### Cyt *b *phylogenetics

**Figure 2:**
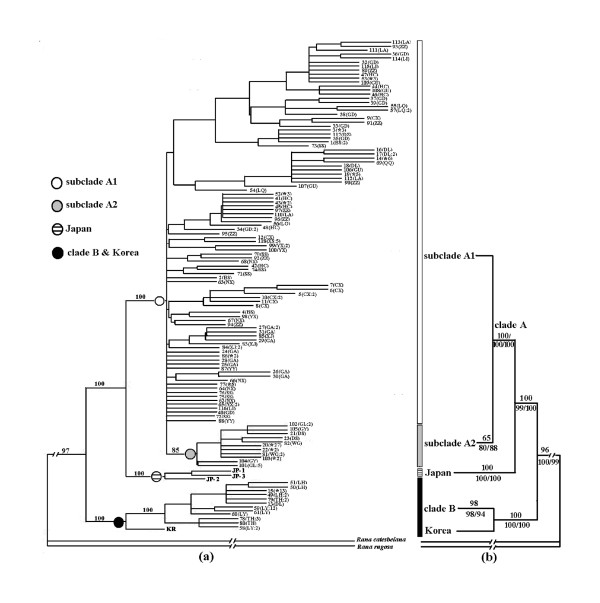
**Phylogenetic trees based on Cyt *b *haplotypes**. (a) Fifty percent majority-rule consensus of 6884 parsimonious trees based on 122 Cyt *b *unique haplotypes of *P. nigromaculata *(CI = 0.7621, RI = 0.9027). Numbers above branches are bootstrap values (1000 replicates). Letters or numbers in parenthesis indicate the population abbreviation and the quantity of individuals sharing the haplotype within each local population. The asterisk followed by an integer indicates the number of individuals sharing the haplotype among different populations (see Table 2 for details). (b) The concordant topology of maximum likelihood, maximum parsimony and neighbor-joining trees resulting from the reduced 49 exemplar ingroup taxa. Numbers above branches are ML bootstrap values (100 replicates); numbers below branches are MP/NJ bootstrap support values (1000 and 5000 replicates).

Maximum parsimony (MP) analysis from 122 ingroup haplotypes resulted in 6884 most parsimonious trees, each 566 steps in length (CI = 0.7621, RI = 0.9027). Two major monophyletic haplotype groups were revealed in the Chinese population of *P. nigromaculata*: designated here as clade A and clade B (as shown in Fig. 2a). Clade A included all haplotypes present in most sampling regions of this frog species: from northeast China (locality 24, 47°20'N) to southwest China (locality 16, 25°08'N); clade B was found limited to eastern part of northeast China (locality 25, 26, 27 and 28). Clade A was further subdivided into two sub-clades: A1, with a broad geographical distribution and weak phylogenetic structure; A2, with higher bootstrap support of 85% and distributed to mountainous regions of southwest China (locality 17–23, see Fig. 1). When included the Korean and Japanese haplotypes, this phylogenetic topology showed that the single Korean haplotype and clade B clustered with 100% bootstrap support, and that the three Japanese haplotypes formed a sister relationship with clade A and showed 100% bootstrap value (Fig. 2).

Maximum parsimony (MP), maximum likelihood (ML) and neighbor-joining (NJ) analysis of our reduced dataset resulted in concordant topologies (topology and bootstrap support value of major clades were shown in Fig. 2b). ML analysis resulted in a tree with a likelihood score of -ln L = 3396.27; MP analysis resulted in 3372 most parsimonious trees, each 528 steps in length (CI = 0.7670, RI = 0.8388). The most obvious feature of the three topologies was consistent with those revealed by MP tree based on 122 haplotypes (Fig. 2a). The sequence distances among these haplotype clades also yielded obvious insight of such phylogenetic relationships (Table 3). The division pattern of haplotypes into several clades (A1, A2, B and Japan) was concordant in three analyses, but support for sub-clade A2 was lower in ML (65%) than in MP (80%) and NJ (88%) analyses. Lower support in ML compared to the MP and NJ analyses was likely the result of a more complex model of sequence evolution used for ML reconstruction.

**Table 3:** Cyt *b *sequence distances and the standard deviations among major lineages under the HKY model.

Lineage	A	A1	A2	JP	B	KR
A	**0.91%**	**----**	**----**			
A1	**----**	**0.90%**				
A2	**----**	1.22% ± 0.002	**0.13%**			
JP	4.06% ± 0.005	4.09% ± 0.005	3.84% ± 0.005	**0.23%**		
B	7.72% ± 0.008	7.07% ± 0.008	7.73% ± 0.008	7.08% ± 0.008	**0.30%**	
KR	7.69% ± 0.008	7.69% ± 0.008	7.71% ± 0.008	6.86% ± 0.008	1.10% ± 0.003	**----**

Bold values along the diagonal are average distance within lineage.

For all the sequence data, Fu and Li's D* and F* were negative and not significant, and Tajima's *D *= -1.22, which was not significantly different from 0 (P > 0.10). Patterns of variation within our mitochondrial data are thus consistent with the neutrality hypothesis.

### PCR-RFLP analysis

Most haplotypes are unique to local populations. Haplotypes shared across local populations (12 instances, Table 2) occur entirely in geographically adjacent areas except for one case (haplotype 3), which is shared between southwest China (BS) and central China (HC). To our surprise, locality 25 (northeast China) is composed of haplotypes grouping of the two highly divergent lineages (A and B). This observation suggests that the Dongliao area (locality 25, DL) may represent a secondary contact zone of the two deeply split lineages. Accordingly, we widened our sampling scales in this region and collected samples from four contiguous sites of 25-a, 25-b, 25-c, and 25-d with the maximum pairwise distances about 28 km (Fig. 1). We used 56 individuals in the PCR-RFLP analysis on Cyt *b*. When the PCR products digested with *Taq *I and *Xba *I, the expected fragment sizes were 1090 and 151 bp for haplotype A, and were 704 and 537 bp for haplotype B. The PCR product-digestion patterns allow us to readily distinguish which haplotype group each product (sample) belongs to. All 12 samples from site 25-a are identified as haplotype A, while 6 and 4 in 25-b are respectively A and B. In 25-c, 7 and 8 belongs to A and B respectively and total 19 samples from 25-d are all B haplotype. Such a distribution pattern strongly supports that secondary contact and introgression between lineages A and B had occurred in this region.

### Nested-clade phylogeographic analysis

**Figure 3:**
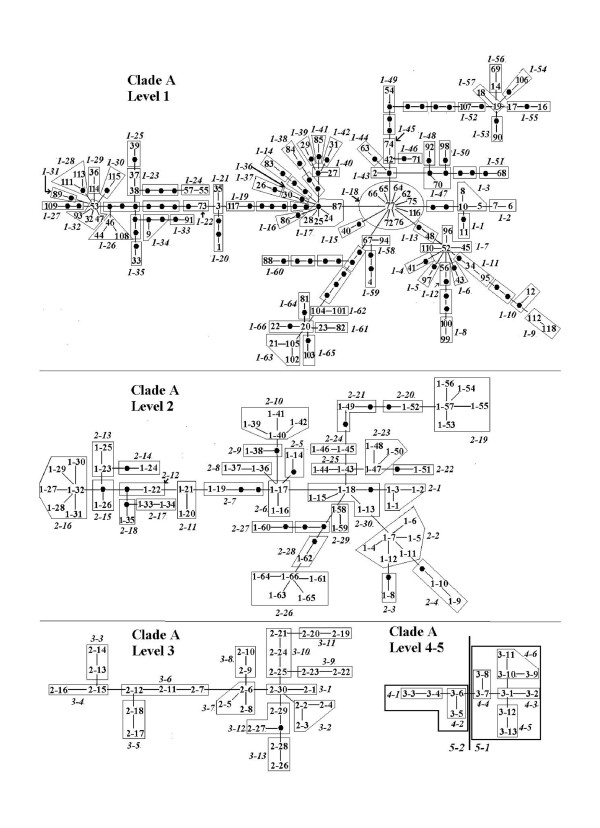
**Nested-clade design on Cyt *b *haplotype parsimony network of *P. nigromaculata *clade A**. Each line represents a single mutational change, irrespective of length. Black circles represent unsampled or extinct haplotypes. Numbers in italic identify clade-groups on each nesting level.

**Figure 4:**
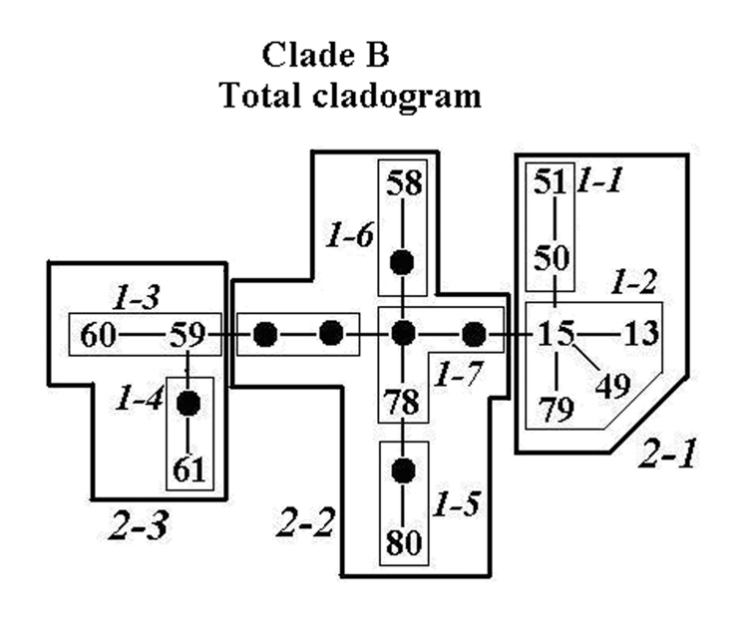
**Nested-clade design on Cyt *b *haplotype parsimony network of *P. nigromaculata *clade B**. Each line represents a single mutational change, irrespective of length. Black circles represent unsampled or extinct haplotypes. Numbers in italic identify clade-groups on each nesting level.

Haplotypes separated by up to eight mutational steps were connected following the hierarchical nesting procedure. The two haplotype clades were grouped into two independent networks (A and B). One hundred and six haplotypes and 5 nesting levels were included in cladogram A (Fig. 3), 12 haplotypes and 2 nesting levels in cladogram B (Fig. 4). For both cladograms, fifteen nested groups showed significant geographical associations in nested contingency test (Table 4). Further, the nested distance analysis showed that 34 nested groups in both haplotype cladograms have geographic distributions that differ significantly from random expectations (results not shown). Contiguous range expansion, isolation by distance, long-distance colonization and past fragmentation were inferred at different levels (Table 5), which facilitated the identification of additional sampling areas.

**Table 4:** Clades with significant associations at the 0.05 level in nested contingency analysis.

Clade	Permutational *χ*^2 ^statistic	Probability
Total cladogram A	95.45	0.000
2-19	37.02	0.015
2-26	65.00	0.000
2-30	23.43	0.046
3-1	44.84	0.000
3-11	14.00	0.003
3-12	9.00	0.018
3-13	18.23	0.029
4-1	52.07	0.002
4-2	20.91	0.000
4-5	73.00	0.000
4-6	39.51	0.000
5-1	101.95	0.000
5-2	50.00	0.000
Total cladogram B	35.65	0.000

For both cladograms, range expansion was inferred at higher levels (Table 5). Tip (young) clades are more widespread geographically than interior (ancestral) haplotypes, which is characteristic of range expansion [18]. For total cladogram A, there was no conclusive inference due to the lack of tip-interior differentiation. Restricted gene flow with isolation by distance is a repeated pattern inferred in clade-groups 2-30, 3-2, 4-1 and 4-2. These four groups correspond to lineage A1 and distribute most sampling localities. For group 3-1 and 3-12 which include haplotypes from coastal population (locality 4), south-western population (locality 16), and most central or eastern populations, we do not have sufficient evidence to discriminate between long-distance movement and the combined effects of gradual movement during a past range expansion and fragmentation. Long-distance colonization (or past fragmentation) is interpreted for group 3-13, which is located in the south-western plateau and corresponds to lineage A2. Inadequate geographical sampling is identified within group 3-11 which corresponds to the regions between northeast and north China (Fig. 1 and 3). Allopatric fragmentation is predicted within group 4-5 and 4-6. The former group is located across southwest China and eastern most regions, while the latter is composed of haplotypes from north-eastern populations and the east China populations.

**Table 5:** Inferences from the nested clade distance analysis in Chinese range of *P. nigromaculata*.

Clade	Inference chain	Inferred pattern
clade A		
5-1 (4)	1-2-11-12-NO	Contiguous range expansion
5-2 (2)	1-19-20-2-11-12-NO	Contiguous range expansion
4-1 (2)	1-2-3-4-NO	Restricted gene flow with isolation by distance
4-2 (2)	1-2-3-4-9-10-NO	Fragmentation or isolation by distance
4-5 (2)	1-19-20-2-3-4-9-NO	Allopatric fragmentation
4-6 (3)	1-2-3-4-9-NO	Allopatric fragmentation
3-1 (2)	1-2-3-5-6-15-NO	Past fragmentation or long-distance colonization
3-11 (2)	1-2-3-5-6-13-14-YES	Range expansion, long-distance colonization or past fragmentation
3-12 (2)	1-2-3-4-9-10-NO	Fragmentation or isolation by distance
3-13 (2)	1-2-3-5-6-13-YES	Long-distance colonization or past fragmentation
2-19 (5)	1-2-3-5-6-13-14-YES	Range expansion, long-distance colonization or past fragmentation
2-30 (3)	1-2-11-17-4-NO	Restricted gene flow with isolation by distance
clade B		
Total cladogram B (3)	1-2-11-12-NO	Contiguous range expansion
1-2 (4)	1-2-11-17-4-NO	Restricted gene flow with isolation by distance

Numbers in parenthesis are amount of sub-level clades within each group.

### Historical demography

**Figure 5:**
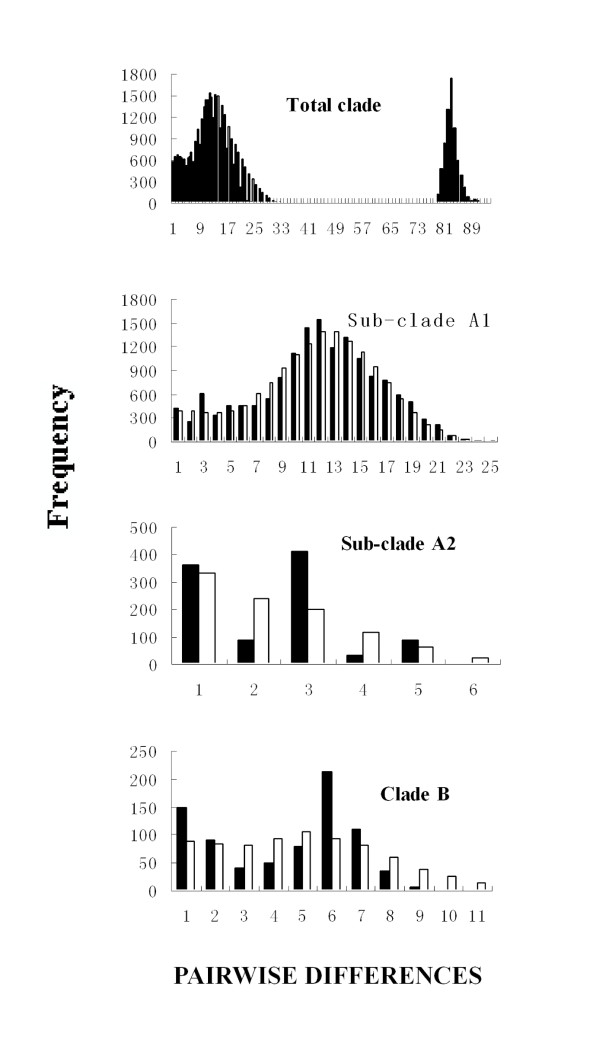
**Mismatch distributions for each major haplotype clade of *P. nigromaculata***. The abscissa shows the number of pairwise differences between compared haplotypes. The ordinate shows the proportion for each value. Black shows observed frequency distribution while the white bars show the distribution expected under the sudden-expansion model.

Mismatch distributions for the entire group exhibited two distinct modes (Fig. 5). We assume that the left peak represents a stationary expansion corresponding to lineage A, but the "older" peak on the right is unlikely due to demographic expansion. Rather, may result from the deep split between the lineages, perhaps caused by bottlenecking and lineage sorting in allopatry (initial reduction of variation). For the three independent lineages, the pairwise differences all show unimodal patterns, which suggests that demographic expansions occurred within each. Mismatch distributions for lineage B and sub-lineage A1 did not differ significantly (*P *> 0.05) from expectations under the sudden-expansion model [19]. Whereas, sub-lineage A2 was weakly significant (*P *= 0.043), but Tajima's *D *and Fu's *Fs *are both negative (Table 6). Our estimated time since expansion for lineage A1, A2 and B were different. Expansions of sub-lineage A1 took place contemporarily about 0.08 Mya, whereas expansion was about 0.012 Mya for sub-lineage A2 and 0.028 Mya for lineage B. The sequence variation (Table 6) of each lineage differs in our estimated h and *π*. The presence of high h and high *π *(0.975 and 2.8%) in the total populations seems to reflect the fact that different mtDNA lineages were analyzed together. Similarly, the same genetic diversity pattern was observed for sub-lineage A1 (h = 0.972,*π *= 0.9%), which means this lineage is a large population with admixed local populations. Relatively low h and extremely low *π *(0.631 and 0.13%) were found in sub-lineage A2, suggesting that a prolonged bottleneck was present in this population. In lineage B, *π *is low (0.3%) but h is otherwise (0.799), which can be explained by rapid population growth from ancestral populations [20].

**Table 6:** Demographic parameters of lineages for which range expansion were detected in mismatch distributions.

Lineage	N_pop_	N_*ind*_	N_hap_	h	*π*	Tajima' *D *(*P*-value)	Fu's *Fs *(*P*-value)
Total population	28	216	118	0.975 ± 0.006	0.028 ± 0.014	-0.097 (0.486)	-23.670 (0.012)
A1	18	121	95	0.972 ± 0.008	0.009 ± 0.005	-1.780 (0.065)	-24.179 (0.000)
A2	7	45	11	0.631 ± 0.080	0.001 ± 0.001	-1.68 (0.043)	-5.040 (0.004)
B	4	40	12	0.799 ± 0.043	0.003 ± 0.002	-0.605 (0.292)	-1.629 (0.247)

N_pop_: number of populations; N_*ind*_: number of individuals; N_hap _number of haplotypes; h: haplotype diversity; *π*: nucleotide diversity.

Based on the results from the MDIV analysis, the posterior distribution for *T *revealed a sharp peak at 1.16. With substitution rate of 4.4% per Myr [21], our estimate of the timing of lineage divergence, 0.92 Mya (95% credibility internal 1.5 to 0.26 Mya), supports a Pleistocene split. Further within the lineage A, divergence time between the two sub-lineages was estimated to be 0.18 Mya (95% credibility internal 0.10 to 0.32 Mya), roughly corresponding to the Riss glaciation (210,000–135,000 years ago) [22].

### ISSR polymorphism and population structure

The 10 ISSR primers generated a total of 127 bands, of which 103 were polymorphic thus showing 81.10% polymorphism and an average genetic diversity of 0.27. The number of bands generated by individual primers varied from 9 to 21, with the average being 12.7. The genetic variation indices at the species level were *PPB *= 81.10, *A*_*o *_= 1.80, *A*_*e *_= 1.45, *H*_*e *_= 0.27, and *I *= 0.40, much higher than the mean values of all populations, especially the *PPB *(data not shown).

**Figure 6:**
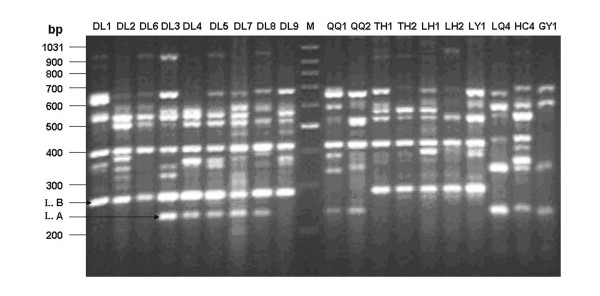
**Amplified patterns generated by using ISSR primer UBC826**. Letters and numbers above the figure are codes for individuals from different localities (letters are abbreviations same to Table 1, numbers are corresponding to individuals). "M" represents the molecular marker of 100-bp ladder, with fragment length of each band indicated. L. A and L. B are bands unique to lineage A and lineage B, respectively.

The UPGMA (Unweighted Pair Group Method with Arithmetic mean) dendrogram showed similar topology to that of the mitochondrial phylogeny, showing divergence between the mitochondrial clade A and clade B, and also between sub-clade A1 and sub-clade A2 (data not shown). As expected, we detected potential gene flow between lineage A and lineage B in DL area. Fig. 6 was the PCR banding pattern with primer UBC826. As shown (right side), band L. A can be readily recognized as a lineage A-special band, while band L. B was unique in lineage B. In the 3 individuals (DL1, 2, 6) of the mitochondrial linage B, L. B was detected, while in 5 individuals (DL3, 4, 5, 7 and 8) of lineage A, both bands (L. A and L. B) were amplified (Fig. 6 left part). Interestingly, in individual DL9 which belongs to mitochondrial lineage A (haplotype 14), only band L. B. was detected. This phenomenon was not found in all other local populations, and provided nuclear evidence to support the secondary contact and introgression between lineage A and B at Dongliao area.

## Discussion

### Evolutionary history and independent refugia

Amphibians are known to exhibit a higher degree of population subdivision than any other major animal taxon [23]. Our analyses revealed that there are two reciprocally monophyletic lineages within Chinese range of *P. nigromaculata*: the mainland lineage (A) and the northeast lineage (B). The deep intraspecific split (7.72%) in Cyt *b *is not of the same magnitude as to some anuran amphibians (maximum of 11.35% in *Mantella bernhardi*; 13.7% in *Hyla arenicolor *[24]) and other vertebrate groups (12.5% in *Microtus oeconomus *[25]). Zhang *et al*. had also detected a deep haplotype split within *P. nigromaculata *on mitochondrial 5' control region [17]. The interpretation was that the relatively long-term hibernation of the north-eastern population limited its ability of gene flow with other mainland populations. In other words, genetic drift under natural selection in northeast China resulted in a deep divergence of this frog species. In such a case, selective sweeps would be expected to affect the evolutionary pattern of Cyt *b*, but this conjecture was not supported by the neutral tests of our dataset which indicated that its patterns of variation were consistent with the hypothesis of neutrality. According to the estimated divergence time of Cyt *b *haplotypes, the two lineages of *P. nigromaculata *diverged approximately 0.92 Mya (credibility internal 1.5 to 0.26 Mya), similar to that of other anurans isolation events [26,27]. This divergence time was roughly congruent with the Gunz glaciation (1.2 – 0.9 Mya), which may be the cause of allopatric isolation and the lineage split.

Geographically, lineage A (A1 and A2) covers almost the entire sampling region of this study. Lineage B is restricted to the eastern part of northeast China (Fig. 1). The patterns of diversification suggest complex histories involving both allopatric isolation among refugial areas and prominent patterns of dispersal. We proposed that there were two independent refugia during Pleistocene glaciations. One refugium was the eastern monsoon region and the lower elevations of the south-western plateau. Unlike Europe and North America, China was not covered by continental ice sheets during the Quaternary [28,29], even though the climate was cold and dry. The permafrost had expanded southward by about 10 degrees latitude to the present day Great Wall line and the ice-age "mammoth fauna" had roamed in north China and had even reached the Yangtze River Estuary [30]. Conversely, the climate in eastern China was relatively moist and warm; therefore, eastern China was likely an alternative for this water-dependent frog to live through the cold periods. Zhang also suggested that eastern China was a refugium for temperate and tropical-subtropical faunas during cold stages of the Quaternary [31]. The fact that most haplotypes from eastern local populations are present in interior clades of the parsimony network A again substantiates our east China refugium contention. Although the south-western plateau had been identified with 3–5 glaciations that are dated to late and middle Pleistocene [32], environmental diversity of tropics and subtropics in lower elevations are still maintained [9]. This region has retained many relic floras of Tertiary and was also a refugium for temperate and subtropical floras in cold periods of Quaternary [33]. It is possible for *P. nigromaculata *to have survived the lower elevations in cold periods and expand afterwards.

We suggest that the other refugium was situated in the present day Korean Peninsula. This Peninsula is situated between 33°–43° N and 124°–132° E, and is adjacent to northeast China. Recent studies on the Quaternary indicated that there were mountain glaciations developed in the Changbai Cordillera [32], which lies between China and North Korea. But the Korean Peninsula, characterized by subtropical mountain climate [10], was not deeply affected by the global climate changes starting in the Pleistocene. The sustained stable environment was presumably favorable to *P. nigromaculata*. The Korean haplotype clustered with clade B and differed by only 0.009 on Cyt *b *sequence from B further strengthens the notion of a Korean refugium.

### Expansion of lineage A

**Figure 7:**
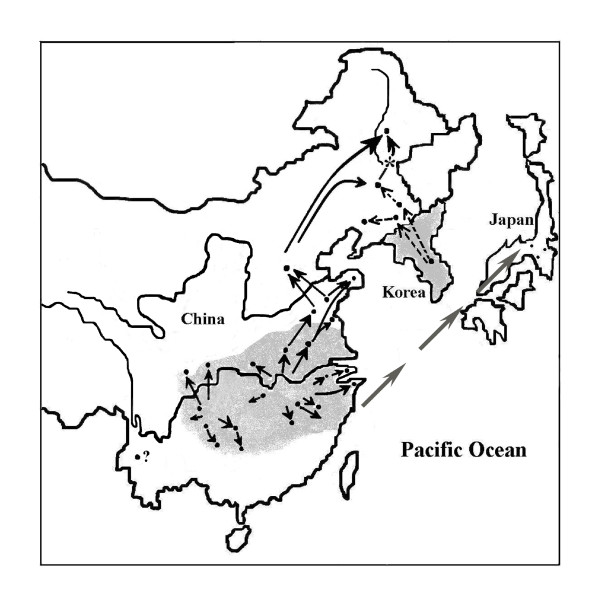
**Map of major dispersal patterns of Chinese population of *P. nigromaculata *identified in this study**. Points show the sampling locations in Fig. 1. Solid arrows indicate dispersal pattern of lineage A. Dotted arrows indicate patterns of dispersal of lineage B. Arrows with star "*" indicate that the pattern is not confirmed. Gray arrows from Chinese eastern coast to Japanese archipelago indicate the dispersal pattern via past land bridge. The symbol "?" indicates that the origin of the population has not been resolved. Gray areas represent past glacial refugia.

Using NCA on the Cyt *b *data, we obtained further insights into the phylogeographic pattern in lineage A of *P. nigromaculata*. First, contiguous range expansion was predicted for cladogram A in the highest nesting level (5-1, 5-2), which represents an older event in the evolutionary history of *P. nigromaculata*. This conclusion helps explain the species' current distribution in the north-eastern areas and in the high elevations of south-western plateau where former glaciations occurred. The tip haplotypes are interpreted as being younger than the interior ones [18]. We found that the majority of younger haplotypes distributed in the most peripheral populations of the species' range (north-eastern, south-western and some costal regions). This pattern, together with the unimodal mismatch distributions, supports the range expansion inference of lineage A. Most ancient haplotypes distributed in eastern China also suggest that this area was the origin of the last interglacial range expansion. From the high genetic variation of this lineage and the fact that most common haplotypes are shared among eastern and south-western populations, we can conclude that the eastern and south-western regions were the ice-age refugium for lineage A. From this refugium, *P. nigromaculata *expanded in all directions to form the current distribution pattern (Fig. 7). Following range expansion, restricted gene flow isolation by distance (IBD) at lower nesting levels (2-30, 3-2, 4-1 and 4-2) took place repeatedly, which seems to represent more recent dynamics of this species. These results likely indicate that gene flow decreased with geographic distance, despite the fact that there were rivers distributed across the frogs' range. It also reflects the reduced vagility of amphibian species. The close relationship between Japanese and clade A haplotypes might be an indication of its origin. Throughout the Quaternary, sea level has risen and fallen as continental ice sheets have waned and waxed [34], and it was about 70–125 m lower than present during the last glacial period when the Eurasia continent was in connection with Japanese archipelago by a land bridge [34-36]. Additionally, the deepest Korean strait makes it controversial of whether a land-bridge connection existed in that place during Late Quaternary [36]. Via the land bridge, we suggest, *P. nigromaculata *colonized Japan from Chinese eastern coast (Fig. 7). However, further samples from the Japanese population are required in future studies.

For group 2-19 (north-eastern populations QQ and DL were included) in lineage A1, we could not distinguish past fragmentation\long-distance colonization from contiguous range expansion due to the inadequate sampling design. Again, in group 3-11, the two north-eastern populations and two populations near the Shandong Peninsula (locality 3, 8) were included, for which the patterns of fragmentation and isolation by distance were not identified due to sampling gaps. Nonetheless, it may imply that, when recolonizing northeast China during the last interglacial period, these peninsula-adjacent populations were founders. The recolonizing route might have been via the Shandong region to northeast China (Fig. 7). This is also consistent with the topology of MP tree (Fig. 2), in which the north-eastern haplotypes are clustered with the peninsula-adjacent haplotypes. The route through which lineage A1 recolonized Baoshan (Location 16) is somewhat puzzlings. The Baoshan population is an isolated population hundreds of kilometres apart from the nearest *P. nigromaculata *population in Sichuan. It is recognized not only by the specimens we obtained in 2002, but also by 63 specimens collected in 1998 by the researchers from Chengdu Institute of Biology, Chinese Academy of Sciences (Fei and Jiang, personal communication). However, due to the relatively high diversity level (both in mitochondrial haplotypes and ISSR profiles), and the relatively few individuals studied, we were unable to unequivocally identify the origin of the Baoshan population.

Group 3-13, corresponding to lineage A2 is composed of all south-western plateau populations characterized by long-distance colonization, and possibly couple with subsequent fragmentation. The unimodal mismatch distribution in this lineage suggests recent rapid colonization. The estimated time since expansion was about 0.012 Mya, after the last glacial maximum (LGM, 0.018 Mya). According to the palaeoclimatic records of this region [9], ice sheet on the mountain had shifted vertically in response to the oscillations of ice ages, natural zones have also moved vertically since the Pleistocene, but retained environmental diversity of tropics and subtropics in lower elevations. *P. nigromaculata *likely survived in these areas in ice-ages and rapidly expanded subsequently. Based on the dispersal theory of Hewitt [2], once a place is occupied by a leading edge expansion population through dispersal, it will be much more difficult for those behind them to follow. The imprint of this is reduced genomic variability. The strikingly low levels of genetic variation, especially the observation that multiple individuals from distant geographic regions share one single haplotype (No. 20 in Table 2: 5 from DS, 3 from WG, 10 from GH, 5 from RH and 4 from DZ) strongly supports rapid expansion.

### Recolonization to northeast China of lineage B

Contiguous range expansion is also inferred for total cladogram B, which restricted to northeast China. Mismatch distribution also lend strong support to the conclusion of population expansion of this lineage. The last global glaciation corresponds respectively to the Würm, Wisconsin and Weichsel glaciation in Alps, North America and western Europe [37]. In China the same period is called Dali glaciation, which began at about 0.05 Mya. In Europe and North America, the most extensive glacial period was during the last glacial maximum (LGM) beginning 0.020 ~ 0.018 Mya, whereas in China the most extensive glacial period was not LGM, but the early stage (0.054 ~ 0.044 Mya) of the Dali glaciation [38] when northeastern China was cold and the Changbai Cordillera was covered with ice caps [32]. The Korean Peninsula was assumed to be a refugium not only because the relatively mild climate of the Peninsula, but also because of the phylogenetic basal position of the Korean haplotype. Our estimated time of expansion was about 0.028 Mya for the *P. nigromaculata *lineage B and it was likely after the early extensive glacial period in Dali glaciation, that this lineage colonized northeast China via the Changbai Cordillera, but not after the last glacial maximum when many lineages in Europe and North America underwent expansions. The current distribution of lineage B suggests that after recolonizing northeast China, this lineage remained *in situ *rather than expanding southward, at least in the present interglacial period.

### Secondary contact and introgressive gene flow

Secondary contact of previously isolated populations after the last glacial maximum in Europe and North-America are well established [2,39-42]. In the present study, we uncovered a population where haplotype lineage A and B of *P. nigromaculata *coexisted in Dongliao area (locality 25). The DL population, located in northeast China, is the only one composed of both lineages, a conclusion confirmed by nucleotide sequences, PCR-RFLP, and ISSR patterns. Hence, the DL region (especially 25-b and 25-c with a distance only 2.2 km) was likely a rather narrow secondary contact zone during the current interglacial period. The full range of the secondary contact zone remains to be defined.

In general, hybrid or contact zones between populations or species of varying degrees of differentiation provide a potentially important source of information about how taxa diverge and populations interact. We could not conclude that in the area of sympatry, genetic isolation exists between the two lineages based solely on high mtDNA sequence divergence of 7.7%. Indeed our nuclear ISSR data suggest some inter-lineage gene flow in the DL population. Two lines of evidence support this contention. First, in 5 individuals of mitochondrial lineage A, both bands of L. A. and L. B. were amplified. Second, one lineage A individual only possessed L. B band. The former may arise from the hybridization between males of lineage B and females of lineage A, and the later might be caused by backcross of female hybrids of the former with males of lineage B. In other words, these two divergent lineages may have experienced a recent hybridization resulting in introgressive gene flow.

## Conclusion

Our phylogeographic study suggests that, two mitochondrial lineages diverged about 0.9 Mya beginning in independent refugia during the Pleistocene glaciation. Two main clades, A and B, differing by *c*. 7.72% sequence divergence were detected. The A clade is further subdivided into two sub-clade, A1 and A2 differing by 1.22%. The two refugia seemed to be located in east China and the lower elevations of south-western plateau, and in Korean Peninsula, respectively. Contiguous range expansion from the two refugia during last interglacial period resulted in the current distribution pattern of this frog species. Lineage A covers almost the entire sampling region of this study. Lineage B is restricted to the eastern part of northeast China. Secondary contact was detected in DL area where both lineages coexisted, as confirmed by nucleotide sequences, PCR-RFLP and ISSR analyses. ISSR results provided further evidence for introgressive gene flow between the two lineages in this area. Although climate events of the Pleistocene in East Asia did not seem to resemble that of Europe or North America, they do have had marked effect on the historical distribution and intraspecific divergence of amphibians as in Europe and North America.

## Methods

### Sample collection and Molecular techniques

Sampling was designed to cover most of the range of *P. nigromaculata *across the temperate and subtropical zones in China. We obtained 262 individuals from 28 localities during the year 2002 and 2003. Two hundred and six samples were used in cytochrome *b *(Cyt *b*) sequence analyses, the other 56, collected from 4 sites (25-a, 25-b, 25-c and 25-d) in Dongliao area (locality 25, DL, Fig. 1) were used in PCR-RFLP analyses to identify secondary contact between haplotype group A and B. The distance between sites 25-a and 25-b is 11.9 km, and that of 25-b and 25-c, 25-c and 25-d is 2.2 and 28.1 km, respectively. For inter simple sequence repeat (ISSR) analyses, six or nine (just in DL) individuals were sampled from the 28 localities mentioned above. *Rana catesbeiana *[GenBank: AF205089] and *Rana rugosa *[GenBank: AF205093] were used as outgroup taxa. Four conspecific haplotypes from Korea (KR) and Japan (JP-1, 2, 3) [GenBank: AF205087, AY315755~AY315757] were also included in the phylogenetic analyses to help infer relationships with Chinese haplotypes. The sampling code and population localities are given in Table 1, and the map of collecting localities is shown in Fig. 1.

Tissue was taken from leg muscle and stored in 95% ethanol. Extraction of DNA followed standard phenol/chloroform extraction methods. The complete 1143 bp of mitochondrial Cyt *b *gene was amplified with primers Glu-f: GAC TCT AAC CTG GAC CAA TAG-3' and contr5'r: TAA ATT TAT GCT CTA TAC A-3', which were designed based on the published mtDNA complete sequence of *Rana nigromaculata *[GenBank: AB043889] [43]. The polymerase chain reaction (PCR) was carried out in 30 μL volumes containing 3.0 μL 10 × Buffer, 2.0 mM MgCl_2_, 0.2 mM each dNTP, 0.25 μM each of primers, 5–10 ng of template DNA and 1.0 U of *Taq *DNA polymerase (Promega). The PCR cycling parameters were 95°C for 4 min, 35 cycles of 95°C for 40 s, 50°C for 45 s, 72°C for 1 min, and 1 cycle of 72°C for 7 min. Templates for sequencing were purified and sequenced in both directions by United Gene Holdings (Shanghai). Nucleotide sequences were initially aligned using CLUSTAL X Version 1.81[44] and corrected manually. Complete sequences were assembled using Seqman II (DNASTAR). The program DNAClub which could predict cutting sites in a DNA sequence was used to search for restriction endonucleases that would yield unique, specific restriction digestion profiles for each haplotype group. For the 56 unsequenced samples, we used two restriction enzymes of *Taq *I and *Xba *I to digest the Cyt *b *products simultaneously, by which we could distinguish the two sharply diverged haplotype groups rapidly and unambiguously.

We also investigated diversification and potential gene flow using inter simple sequence repeat PCR (ISSR). Ten 3'-anchored primers (UBC807, 808, 809, 811, 817, 818, 825, 826, 827 and 835) from University of British Columbia (ISSR set #9) were used for the study. The reaction mixture (25 μL) contained 2.5 μL of 10 × reaction buffer, 25 ng of DNA template, 0.5 μM of a single primer, 2.0 mM MgCl_2_, 0.2 mM of each dNTP and 1.0 U of *Taq *DNA Polymerase (Promega). The PCR products were separated on 2% agarose gels buffered with 0.5 × TBE, detected by staining with ethidum bromide, and photographed under ultraviolet light. Molecular weights were estimated using a 100-bp DNA ladder.

### Phylogenetic analysis

We used PAUP*4.0b10 [45] for phylogenetic analyses using maximum parsimony (MP), maximum likelihood (ML) and neighbor-joining (NJ) methods. Most local populations showed low level of genetic differentiation and were composed of haplotypes with only 1 to 3 nucleotide changes. For ML phylogenetic reconstruction, these similar haplotypes would considerably increase the computation time. Therefore, the dataset comprised of all haplotypes was only used for MP reconstruction. To save the computing time, we cut the sequences with less than four-step substitutions for ML analysis to a 49 ingroup exemplar dataset including 45 from China, 1 from Korea and 3 from Japan. The best-fit likelihood model of sequence evolution was HKY + I + G, which was chosen on the basis of hierarchical likelihood-ratio tests (hLRTs) as implemented in MODELTEST 3.06 [46]. The parameters of the model were calculated using PAUP*. The base frequencies were 0.2582, 0.3340, 0.1334 and 0.2743 for A, C, G and T, respectively, transition/transversion ratio ti/tv = 4.2823, proportion of invariable sites Pinvar = 0.5841, gamma distribution shape parameter α = 1.9961. We also conducted MP and NJ analyses for our reduced exemplar taxa. ML heuristic search was conducted using the following options: addition sequence = 'as-is', with 1 tree held at each step, TBR swapping algorithm, Collapse and Multrees options in effect, steepest descent option not in effect. Nodal support of the ML tree was estimated using bootstrap method [47] with 100 pseudoreplicates; Maximum parsimony (MP) analyses were conducted using heuristic search with 100 random sequence additions and tree-bisection-reconnection (TBR) branch swapping. Robustness of the MP trees was assessed by 1000 bootstrap replicates; NJ reconstruction was conducted using distances calculated under the HKY model, support for the topology was evaluated by bootstrapping 5000 replicates. We also used the chosen model to calculate the diversity indices and the sequence distances.

We calculated the statistical tests D* and F* proposed by Fu and Li [48], and the *D *test statistic proposed by Tajima [49] implemented in DnaSP 3.0 [50] for testing the hypothesis that all mutations are selectively neutral [51].

### Nested-clade phylogeographic analysis

We applied nested clade analysis (NCA [18,52]) to gain further insight into the demographic history of *P. nigromaculata*. The statistical parsimony [52] cladogram networks were constructed using TCS version 1.13 [53]. The connection for haplotypes at 95% confidence level were nested into higher level clades followed the algorithm given by Templeton *et al*. [52]. We used GEODIS version 2.0 [54] to test the null hypothesis of no association between haplotypes and their geographical locations by randomly permuting the observations within a nesting clade across geographical locations. After 1000 random permutations, the clade and nested clade distances (D_*c *_and D_*n*_), the interior-tip distances (I-T_*c *_and I-T_*n*_) were calculated. The observed D_*c*_and D_*n *_were then contrasted to the null distribution to infer the statistically significantly large (L) and small (S) distances at 5% deviation level. For the clades with significant geographical associations, the recent published inference key [55] was used to infer possible causation.

### Demographic history

Mismatch distributions were created using ARLEQUIN version 2.000 [56] to detect historical demographic expansions for the major lineages (with or without range expansion in NCA analysis). We also calculated Tajima's *D *[49,57] and performed Fu's *Fs *test [58] to confirm evidence of demographic expansion. We estimated the time since expansion with the equation τ = 2*ut *[19], where τ is the mode of the mismatch distribution, an index of time since expansion expressed in units of mutational time, *u *is the mutation rate for the whole sequence, and *t *is the time since expansion. Finally, the comparisons of haplotype diversity (*h*) and nucleotide diversity (*π*) within different lineages were used to infer historical demography and examine the biological inference from our nested clade analysis.

### Divergence time between lineages

We analyzed the divergence time between the two lineages with a coalescent-based approach using the program MDIV [59]. We ran the program under the 'finite model', which does not assume that only one mutation occurs at each site but takes into account the possibility of multiple hits, differences in the nucleotide frequencies and the presence of transition/transversion bias. MDIV estimated values for theta (*θ *= 4*N*_*e*_μ), migration rate (*M *= 2*N*_*e*_m), time of divergence (*T *= t/2*N*_*e*_) [59]. We then ran each simulation 5 × 10^6 ^times with a 10% burn- in period as suggested. Likelihood values for *θ*, *M *and *T *were calculated and the value with the highest posterior probability accepted as the best estimate. Values for *T *were calculated using an evolution rate estimated at 4.4% per Myr [21].

### ISSR analysis

Unequivocally scorable and consistently reproducible amplified ISSR bands were scored as present (1) and absent (0), each of which was treated as an independent character regardless of its intensity. Fragments of the same molecular weight were considered as the same locus.

The binary matrix was used under the Hardy-Weinberg equilibrium to calculate the percentage of polymorphism (*PPB*), Nei's gene diversity (*H*_*e*_), the observed number of alleles per locus (A_*o*_), the effective number of alleles per locus (A_*e*_), Shannon's information index (*I*), total gene diversity, the level of gene flow using POPGENE version 1.31 [60].

Furthermore, to illustrate the relationship among populations, genetic divergence (*F*_*st*_) were calculated using software ARLEQUIN version 2.000. We generated UPGMA (unweighted pair group method with arithmetical averages) trees using the SAHN- clustering and TREE programs from the NTSYS-pc, version 2.1 package [61]. In this tree, individuals were enforced to their populations except for those from the DL population (site 25), which were subdivided into DLA and DLB according to their ISSR patterns.

## Abbreviations

ISSR: Inter simple sequence repeat; PCR-RFLP: Polymerase chain reaction-restriction fragment length polymorphism; MP: Maximum parsimony; ML: Maximum likelihood; NJ: Neighbor-joining; UPGMA: Unweighted Pair Group Method with Arithmetic mean; NCA: Nested clade analysis; IBD: Isolation by distance; LGM: Last glacial maximum.

## Authors' contributions

HZ isolated genomic DNA, and conducted the amplification and following RFLP analysis of the complete Cyt *b *gene. She collected, analyzed and summarized the data, and drafted the manuscript; JY participated in analysis and interpretation of data, and helped draft the manuscript; GQZ performed ISSR analysis; KYZ conceived the study and participated in its design and data interpretation, and preparing the manuscript. All authors read and approved the final manuscript.
